# DNA Methyltransferase 3b Accelerates the Process of Atherosclerosis

**DOI:** 10.1155/2022/5249367

**Published:** 2022-04-05

**Authors:** Ling Zhu, Lei Jia, Na Liu, Runmiao Wu, Gongchang Guan, Rutai Hui, Yujie Xing, Yong Zhang, Junkui Wang

**Affiliations:** ^1^Department of Cardiology, Shaanxi Provincial People's Hospital, Xi'an, Shaanxi 710000, China; ^2^Department of Cardiology, The Third Affiliated Hospital of Xi'an Jiaotong University, Xi'an, Shaanxi 710000, China; ^3^Department of Cardiology, State Key Laboratory of Cardiovascular Disease, Fuwai Hospital, National Center for Cardiovascular Diseases, Chinese Academy of Medical Sciences and Peking Union Medical College, Beijing 100037, China; ^4^Department of Pediatric Cardiology, Shaanxi Provincial People's Hospital, Xi'an, Shaanxi 710000, China; ^5^Department of Respiratory and Critical Care Medicine, Shaanxi Provincial People's Hospital, Xi'an, Shaanxi 710000, China

## Abstract

**Background:**

DNA methylation plays a key role in establishing cell type–specific gene expression profiles and patterns in atherosclerosis. The underlying mechanism remains unclear. Previous studies have shown that DNA methyltransferase 3b (DNMT3b) may play an important role in atherosclerosis. This study aimed to establish the regulatory role of DNMT3b in the development of atherosclerosis.

**Methods:**

We constructed a viral vector carrying Dnmt3b shRNA to transduce ApoE^−/−^ mice. Meanwhile, healthy human peripheral blood Treg cells were treated with inhibitor of DNMT3b (AZA and EGCG) or transduced with DNMT3b shRNA.

**Results:**

It showed that Dnmt3b silencing attenuated atherosclerosis, including decreased lesion size and macrophage content and increased collagen and smooth muscle cells content in ApoE^−/−^ mice. To further investigate the possible mechanisms, combined with previous studies by our group, we showed that *Foxp3*-TSDR methylation level was significantly reduced Foxp3 expression and peripheral blood Treg levels were significantly increased by Dnmt3b shRNA vector transduction in animals committed to western diet for 12 and 18 weeks. Consistently, inhibition of DNMT3b (AZA and EGCG) decreased the expression levels of DNMT3b, which can increase the expression levels of FOXP3, and increase the levels of TGF-*β* and IL-10 and decrease the levels of IL-*β* and IFN-*γ*. After transduction with DNMT3b shRNA, the effect was more obvious.

**Conclusions:**

DNMT3b accelerated atherosclerosis, and may be associated with FOXP3 hypermethylation status in regulatory T cells.

## 1. Introduction

Atherosclerosis is a chronic inflammatory disease of the arterial vessel wall [1]. Multiple studies have pointed out that inflammation is the common link of multiple risk factors for atherosclerosis [2]. Recent investigations on etiopathogenesis pointed out alterations of several inflammation-related DNA methylation were highly associated with atherosclerosis [3–5].. DNA methylation and its regulation play critical roles in establishing cell type-specific gene expression profiles and patterns [6–8]. For instance, Dunn et al. showed that hypermethylation of HoxA5 and Klf3 contributed to endothelial cell inflammation and induces atherosclerosis [4]. Moreover, an increase of DNA methylation in cellular repressors of E1A-stimulated genes suppresses its expression and contributes to endothelial dysfunction and atherosclerosis development [9]. Results from these previous investigations indicated that DNA methylation dysregulation of certain genes takes responsibility in the occurrence and development of atherosclerosis.

DNA methylation happens almost exclusively on the cytosine residue within CpG doublet, which depends on DNA methyltransferanses (DNMTs). It is now accepted that DNMT3a and DNMT3b are responsible for the de novo methylation mode, whereas DNMT1 is responsible for maintaining methylation [10, 11]. It has been found that DNMT3b expression is associated with the development of atherosclerosis [12, 13]. However, the role and mechanism of action of DNMT3b in regulating DNA methylation are poorly understood.

According to our previous study, the expression of DNMT3b, rather than other DNMTs, increased significantly in a specific subgroup of T cells, namely, regulatory T cells (Tregs) extracted from patients with coronary artery disease and isolated peripheral blood of healthy volunteers [14]. Our further investigation showed that elevated methylation levels of *FOXP3* are associated with atherosclerosis by reducing the percentages of Tregs [15]. Thus, we hypothesize that DNMT3b-mediated hypermethylation of FOXP3 in Tregs would be one of the underlying mechanisms of atherosclerosis.

## 2. Materials and Methods

### 2.1. Animals

ApoE^−/−^ mice (C57BL/6J background) were weaned at 4 weeks old and were fed with a standard chow diet until 6 weeks of age and then were switched to a western diet (WD) containing 21% fat and 0.15% cholesterol. Twenty-four male mice (6 weeks old) were randomly assigned to 4 groups (12 weeks WD + scramble control; 12 weeks WD + Dnmt3b shRNA; 18 weeks WD + scramble control; 18 weeks WD + Dnmt3b shRNA, *n* = 6). Tail vein injection was performed at the 6, 9, and 12 weeks old, respectively. The 12 weeks groups were fed a WD for a period of 12 weeks (from 6 to 18 weeks old) and received rLV-shRNA-mDnmt3b or rLV-null delivery by tail vein injection at 6 weeks and 9 weeks old, respectively (200 *μ*l, 5 × 10^7^TU/ml). The 18 weeks groups were fed with WD for 18 weeks (from 6 to 24 weeks old) and received rLV-shRNA-mDnmt3b or rLV-null delivery by tail vein injection at 6 weeks, 9 weeks, and 12 weeks old, respectively (200 *μ*l, 5 × 10^7^TU/ml). The diagram of animal experiments is presented in Figure S1 (supplementary files). Animals were anesthetized by isoflurane inhalation (3% for introduced and 1.5% for continuous anesthesia) at 0.4 L/min and fixed in a supine position, collected peripheral blood, euthanized immediately through cervical dislocation, then the animals were perfusion fixed, removed the spleen and aortic root. All animal studies were performed in accordance with the Recommended Guideline for the Care and Use of Laboratory Animals issued by Chinese Council on Animal and were approved by the Medical Animal Research Ethics Committee at Xi'an Jiaotong University.

### 2.2. Flow Cytometric Analysis of Tregs

The percentage of CD4^+^CD25^+^Foxp3^+^ Tregs/CD4^+^ T cells in total peripheral blood of ApoE^−/−^ mice was analyzed by using an eBioscience Mouse Regulatory T Cell Staining Kit (FOXP3 FJK-16s PE, CD4 FITC, CD25 APC, Catalog No. 88-8111-40; eBioscience) with a flow cytometer (BECKMAN COULTER) according to the protocol provided by the manufacturer. We first gated the CD4^+^ T cells and then gated CD25^+^Foxp3^+^ in CD4^+^ cells (Figure S2 supplemental files). Analysis was carried out on a CytoFLEX with CytExpert software (BECKMAN COULTER).

### 2.3. Atherosclerotic Lesions Evaluation

The aortic root was dissected under a microscope and frozen in optimal cutting temperature embedding medium for serial cryosectioning at 10 *μ*m, covering 400 *μ*m of the aortic root. The first section was collected when 3 aortic valve cusps became visible in the lumen of the aorta. Every tenth section was harvested on one slide (2 sections per slide). Lipids were detected by Oil Red O staining (Sigma). Collagen was stained with Sirius Red (Sigma). Section images of lipids were captured digitally by an Olympus BX53 imaging system (Olympus, Tokyo, Japan), and section images of collagen were captured digitally by Nikon Eclipse ci imaging system (Nikon, Tokyo, Japan). Macrophage content was analyzed by immunofluorescence staining with CD68 monoclonal antibody (CD68, 1 : 100, Abcam). Immunofluorescence staining was performed with primary antibodies to identify smooth muscle cells (*α*-SMA, 1 : 100, Abcam). Then, the sections were further incubated with secondary antibodies conjugated with Alexa 555 (Abcam). Nuclei were tagged with DAPI. The images were visualized and captured by using a microscope (Olympus, BX53).

### 2.4. Construction, Packaging, and Purification of pLVX-shRNA1-mDNMT3b

#### 2.4.1. Construction of pLVX-shRNA1-mDNMT3b

The mice Dnmt3b mRNA sequence was obtained from GenBank. The sequence was 5′-GCATGAAGGCCAGATCAAA-3′, which was designed and synthesized by GenePharma (Shanghai, China). The scrambled sequence was 5′-UUCUCCGAACGUGUCACGU-3′. BamHI and EcoRI enzymes (NEB) were used to digest and linerize the vector pLVX-shRNA1. The structure of the vector was demonstrated in Figure S3A (supplemental files). The products were mixed with competent cells JM109 which were further cultured on LB medium containing Ampicillin. A Plasmid Preparation kit (Beyotime) was used to extract the recombinant plasmids which were further identified by XhoI enzyme (NEB) digestion and DNA sequencing.

#### 2.4.2. Packaging and Purification of pLVX-shRNA1-mDNMT3b

293 T cells were cultured with DMEM supplemented with 10% FBS at 37°C in a humidified atmosphere providing 5%CO2 and 95% air. The pLVX-shRNA1-mDNMT3b plasmids were transduced into the cells with calcium phosphate method when the confluence of T293 cells reached 60%. Observation of fluorescence was considered the confirmation of successful transduction (Figure S3B, supplemental files). 72 hours after packaging, the viral supernatant was collected by centrifugation. The vectors were further purified by using Lenti-Pac Lentivirus Concentration Solution (GeneCopoeia, LT007). qPCR was used to determine the viral titer.

#### 2.4.3. Cell Isolation and Treatment

Peripheral blood CD4^+^CD25^+^ Tregs were collected and isolated from one healthy volunteer by magnetic cell separation using CD4+CD25+ Regulatory T Cell Isolation Kit (Miltenyi Biotec, Bergisch Gladbach, Germany) according to our previous descriptions [14]. Isolated CD4+CD25+ Tregs were cultured and stimulated based on previously reported protocols with minor modifications [16, 17]. Isolated CD4+CD25+ Tregs were cultured at a density of 2.5 × 10^5^ cells per well in a 24-well culture plate supplemented with soluble anti-CD3 (1 mg/ml), IL-2 (100 U/ml), and 50 *μ*g/ml fresh oxidized low-density lipoprotein (ox-LDL) (Peking Union-Biology, Beijing, China). In inhibition experiments, Tregs were stimulated with inhibitors of DNA methyltransferases (5-aza-2′-deoxycytidine, AZA [10 *μ*M] and epigallocatechin-3-gallate, EGCG [40 *μ*M]) (Sigma-Aldrich, St. Louis, MO, USA) and were transduced with DNMT3b shRNA (multiplicity of infection =25), respectively. Tregs were cultured for 72 hours in an atmosphere of 5% CO2 at 37°C. The experiments were repeated three times and each was from one healthy volunteer. The healthy volunteer gave informed consent to participate in this study, which was approved by the Ethics Committee of Xi'an Jiaotong University.

#### 2.4.4. Bisulfite Sequencing

The methylation of *Foxp3* gene TSDR from mouse peripheral blood mononuclear cells was determined by bisulfite sequencing as described in our previous study [14]. Primer sequences of *Foxp3*-TSDR methylation and *Foxp3*-TSDR sequences of mice are listed in Table S1 (supplemental files).

#### 2.4.5. Quantitative RT-PCR

Total RNA (isolated CD4+CD25+ Tregs of human peripheral blood and mouse spleen tissue) was isolated using Trizol (Aidlab Biotechnologies, Beijing, China) according to the protocol provided by the manufacturer. PrimeScript RT reagent Kit (TaKaRa Biotechnology, Dalian, China) was used to synthesize the cDNA. The RT-PCR was carried out by using SYBR green method (Applied Biosystems, Foster City, CA, USA). GAPDH was used as an internal control for normalizing expression. Samples were run for 95°C for 10 min, followed by 40 cycles of 30 sec at 95°C and 30 sec at 60°C. Relative expression levels were analyzed by the 2—△△Ct method. Sequence of quantitative RT-PCR was indicated in Table S2 (supplemental files).

### 2.5. Western Blotting

Isolated human CD4+CD25+ Tregs from peripheral blood and mouse CD4+CD25+ Tregs from spleen tissue were homogenized and lysed by RIPA lysis buffer system (Santa Cruz). Total protein was quantified with a BCA kit (Invitrogen). 40 *μ*g protein sample was subjected to SDS-PAGE. Separated protein was then blotted onto PVDF membranes, which were then blocked with blocking buffer (Beyotime) at room temperature for 2 h before incubation with specific antibodies (DNMT3b, 1 : 2000, Abcam; FOXP3, 1 : 1000, Abcam; GAPDH, 1 : 1000, Abcam). The membranes were incubated at 4°C for 10 h and then washed by TBST for five or six times. Subsequently, the membranes were incubated with HRP-conjugated secondary antibodies (1 : 50000, Abcam) at 37°C for 2 h and then washed by TBST for five or six times. The membranes were developed by ECL kit (Thermo) and were detected by Gene Genius System (Syngene). The band intensities were measured using BandScan software.

### 2.6. Enzyme-Linked Immunosorbent Assay (ELISA)

Contents of TGF-*β*, IL-10, IL-1*β*, and IFN-*γ* in culture medium of isolated Tregs were measured by using commercially available ELISA kits (Elabscience, Wuhan, China. Both the inter-assay and the intra-assay coefficient of variation of TGF-*β*, IL-10, IL-1*β*, and IFN-*γ* were <10%). Plasma of ApoE^−/−^ mice was separated by centrifugation and stored at −80°C. Plasma concentrations of TGF-*β*, IL-10, and ox-LDL in ApoE^−/−^ mice were measured using ELISA kits according to the manufacturers' instructions (TGF-*β* and IL-10 kit from Elabscience, Wuhan, China. Both the inter-assay and the intra-assay coefficient of variation were <10%; ox-LDL kit from Cloud-Clone Corp., Wuhan, China. The inter-assay coefficient of variation was <12%, and the intra-assay coefficient of variation was <10%).

### 2.7. Statistical Analysis

All data were expressed as means±SE. The statistical significance of the groups was analyzed by *t*-test or Mann–Whitney *U* test. A level of *P* < 0.05 was considered statistically significant. All analyses were performed with PASW Statistics 20.0 software.

## 3. Results

### 3.1. Dnmt3b Silencing Attenuated Atherosclerosis in ApoE^−/−^ Mice

**Figure 1:**
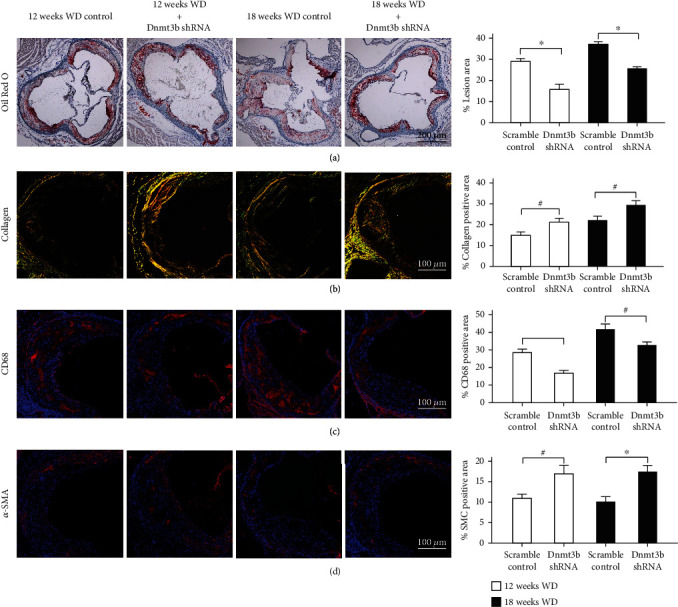
Effects of Dnmt3b silencing on aortic root atherosclerosis lesions in ApoE^−/−^ mice. ∗*P* < 0.01, #*P* < 0.05. (a) Left: The fraction area of lesion was measured by Oil Red O staining, magnification ×40. Right: Quantitative analysis of aortic root AS lesion area. (b) Left: The collagen content was assessed by Sirius Red staining, magnification ×100. Right: Quantitative analysis of collagen content of plaques (3 sections per mice, *n* = 6). (c) The macrophage (CD68) content was evaluated by immunofluorescence staining, magnification ×100. Right: Quantification of macrophage content in lesion by measuring the CD68-positive area within lesion (3 sections per mice, *n* = 6). (d) The smooth muscle cells (*α*-SMA) content was evaluated by immunofluorescence staining, magnification ×100. Right: Quantitative analysis of smooth muscle cells content in lesion by measuring the *α*-SMA-positive area within lesion (3 sections per mice, *n* = 6). Abbreviations: Dnmt3b: DNA methyltransferases 3b; WD: western diet.

We constructed a viral vector carrying Dnmt3b-shRNA which was used to transduce ApoE^−/−^ mice via tail vein injections. Dnmt3b-shRNA vector transduction showed a significant attenuating effect on aortic root plaque area in animals committed with WD for 12 and 18 weeks compared with controls (Figure 1(a)). The lesion area of the aortic root is presented in Figure S4 (supplemental files). The macrophage content (Figure 1(c)) was also markedly decreased in plaques of the aortic root area of ApoE^−/−^ mice committed to WD for 12 and 18 weeks. The collagen content (Figure 1(b)) and smooth muscle cell content (Figure 1(d)) were significantly increased compared with control. These results suggest that knockdown Dnmt3b ameliorated the development of accelerated AS and stabilized plaques.

### 3.2. Dnmt3b Silencing Increased Percentages of Treg and Suppressed Inflammatory in Peripheral Blood of ApoE^−/−^ Mice

**Figure 2:**
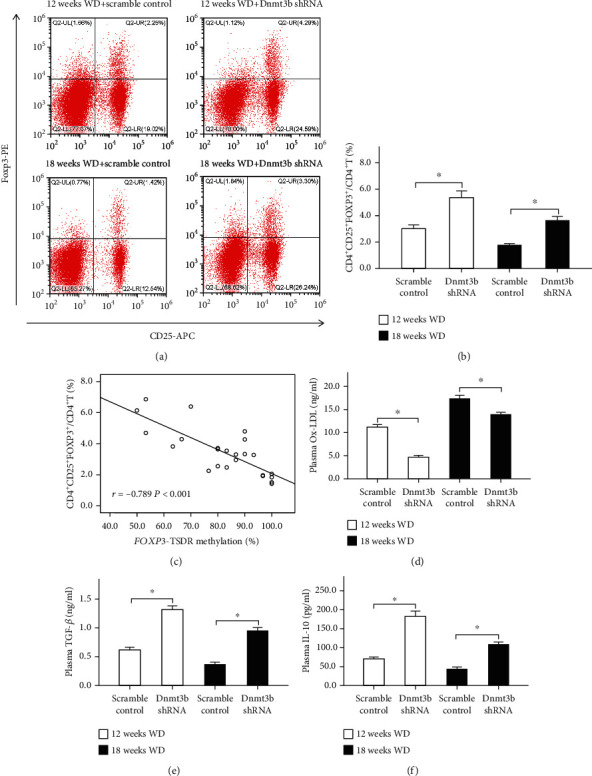
The percentages of Treg and inflammatory cytokine levels in peripheral blood of ApoE^−/−^ mice. ∗*P* < 0.01. (a) Representative FACS results of Tregs (gated by CD4^+^ T cells) from a single mouse in four groups. Numbers represent the percentage of cells in the quadrants (CD4^+^CD25^+^Foxp3^+^/CD4^+^ T cells). (b) Results of the statistical analysis of peripheral blood Treg among four groups. (c) The correlation between the methylation levels of *Foxp3*-TSDR and Tregs ratio of peripheral blood in ApoE^−/−^ mice was calculated by using the Spearman rank correlation coefficient (*r* = −0.789, *P* < 0.001). (d) The plasma concentrations of ox-LDL among four groups. (e) The plasma concentrations of TGF-*β* among four groups. (f) The plasma concentrations of IL-10 among four groups. Abbreviations: Foxp3: forkhead box P3; ox-LDL: oxidized low-density lipoprotein; TSDR: Treg-specific demethylated region; WD: western diet.

As the methylation levels of *Foxp3*-TSDR decrease, the percentages of Tregs (CD4^+^CD25^+^Foxp3^+^/CD4^+^) increased accordingly in 12 weeks and 18 weeks WD combined with Dnmt3b shRNA, respectively (Figures 2(a) and 2(b)). Moreover, we found that Treg levels were significantly inversely correlated with the methylation levels of Foxp3-TSDR in peripheral blood of ApoE^−/−^ mice (*r* = −0.789, *P* < 0.001; Figure 2(c)). Compared with controls, the plasma concentration of ox-LDL was significantly reduced by Dnmt3b-shRNA vector transduction (Figure 2(d)) in animals committed to WD for 12 and 18 weeks. Plasma concentrations of TGF-*β* (Figure 2(e)) and IL-10 (Figure 2(f)) were significantly upregulated in animals committed to WD for 12 and 18 weeks compared with controls.

### 3.3. Dnmt3b Silencing Enhanced Foxp3 Expression of Treg by Reducing *Foxp3*-TSDR Methylation Levels in ApoE^−/−^ Mice

**Figure 3:**
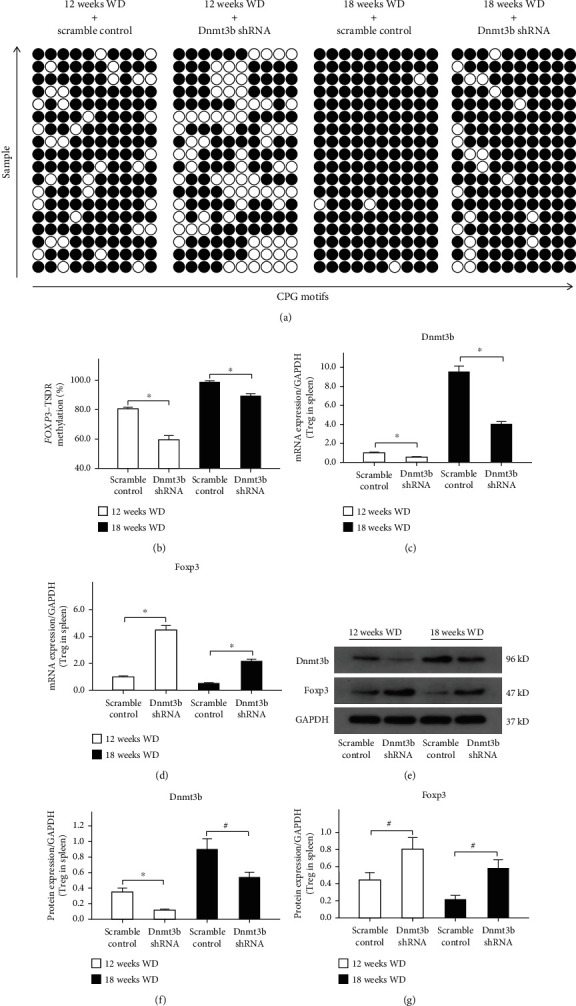
*Foxp3*-TSDR methylation levels of peripheral blood and FOXP3 expression levels of Treg in ApoE^−/−^ mice. (a) Methylation status of 10 individual CpG motifs of the *Foxp3*-TSDR is shown by white (demethylation) or black (methylation) circles. Repeat sequencing for 3 times per mouse. (b) Results of the statistical analysis of *Foxp3*-TSDR methylation (*n* = 6, repeated 3 times). ∗*P* < 0.01, #*P* < 0.05. (c) The mRNA expression levels of Dnmt3b in spleen Treg after transduction Dnmt3b-shRNA into ApoE^−/−^ mice (*n* = 6, repeated 3 times). (d) The mRNA expression levels of Foxp3 in spleen Treg after transduction Dnmt3b-shRNA into ApoE^−/−^ mice. (e) Western blot results of Dnmt3b and Foxp3 in spleen Treg after transduction Dnmt3b-shRNA into ApoE^−/−^ mice (*n* = 6, repeated 3 times). (f) The protein expression levels of Dnmt3b in spleen Treg after transduction Dnmt3b-shRNA into ApoE^−/−^ mice. (g) The protein expression levels of Foxp3 in spleen Treg after transduction Dnmt3b-shRNA into ApoE^−/−^ mice. Abbreviations: Dnmt3b: DNA methyltransferases 3b; Foxp3: forkhead box P3; TSDR: Treg-specific demethylated region; WD: western diet.

CD4+CD25+ Tregs of the spleen were collected and isolated from ApoE^−/−^ mice by magnetic cell separation using CD4+CD25+ Regulatory T Cell Isolation Kit. After transduction Dnmt3b-shRNA into ApoE^−/−^ mice, the expression level of Dnmt3b of Treg was significantly decreased in the spleen of ApoE^−/−^ mice (Figures 3(c), 3(e), and 3(f)). Compared with the control, Dnmt3b-shRNA vector transduction decreased *Foxp3*-TSDR methylation levels in peripheral blood of ApoE^−/−^ mice after mice were committed to WD 12 and 18 weeks (Figures 3(a) and 3(b)). As the methylation levels of *Foxp3*-TSDR decrease, the Foxp3 mRNA (Figure 3(d)) and protein (Figures 3(e) and 3(g)) expression levels were also enhanced in Treg in the spleen of ApoE^−/−^ mice. Compared with the 12 weeks WD control, 12 weeks WD combined with Dnmt3b shRNA increased Foxp3 mRNA and protein expression levels of Treg in the spleen of ApoE^−/−^ mice. The Foxp3 mRNA and protein expression levels of Treg were also significantly upregulated in 18 weeks WD combined with Dnmt3b shRNA in the spleen, compared with 18 weeks WD.

### 3.4. DNMT3b Inhibition Increased FOXP3 Expression in Ox-LDL Cultures of Isolated Tregs

Healthy human peripheral blood Treg cells stimulated by ox-LDL were treated with inhibitors of DNA methyltransferases (AZA and EGCG) or transduced with DNMT3b shRNA (peripheral blood CD4+CD25+ Tregs were collected and isolated by magnetic cell separation using CD4+CD25+ Regulatory T Cell Isolation Kit).

**Figure 4:**
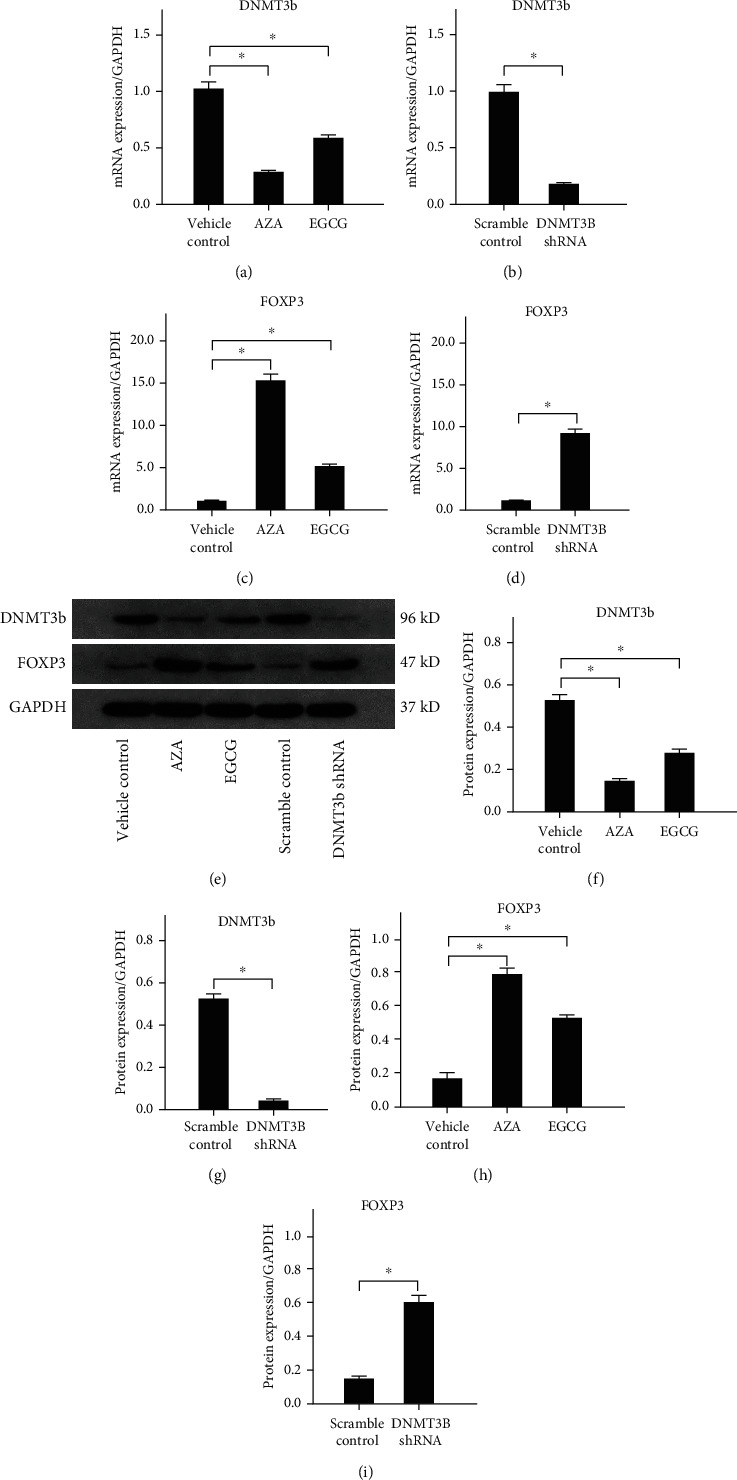
The expression levels of FOXP3 after down-regulation of DNMT3b in ox-LDL cultures of isolated Tregs. ∗*P* < 0.01. (a) The mRNA expression levels of DNMT3b by stimulating with AZA and EGCG. (b) The mRNA expression levels of DNMT3b by transduced with DNMT3b shRNA. (c) The mRNA expression levels of FOXP3 by stimulating with AZA and EGCG. (d) The mRNA expression levels of FOXP3 by transduced with DNMT3b shRNA. (e) Western blot results of DNMT3b and FXOP3 after down-regulation of DNMT3b. (f) The protein expression levels of DNMT3b by stimulating with AZA and EGCG. (g) The protein expression levels of DNMT3b by transduced with DNMT3b shRNA. (h) The protein expression levels of FOXP3 by stimulating with AZA and EGCG. (i) The protein expression levels of FOXP3 by transduced with DNMT3b shRNA. Abbreviations: AZA: 5-aza-2′-deoxycytidine; DNMT3b: DNA methyltransferases 3b; EGCG: epigallocatechin-3-gallate; FOXP3: forkhead box P3; ox-LDL: oxidized low-density lipoprotein.

Compared with the vehicle control, AZA and EGCG decreased the mRNA and protein expression levels of DNMT3b (Figures 4(a), 4(e), and 4(f)). The transduction of DNMT3b shRNA also reduced the expression level of DNMT3b in Tregs (Figures 4(b), 4(e), and 4(g)). As the expression levels of DNMT3b decreased (stimulated with AZA and EGCG), the mRNA level of FOXP3 was significantly increased (Figure 4(c)). FOXP3 mRNA level was also significantly increased in ox-LDL-treated Tregs after DNMT3b shRNA transduction (Figure 4(d)). FOXP3 protein expression alterations were consistent with the mRNA expression after stimulated with AZA and EGCG (Figures 4(e) and 4(h)) or transduced with DNMT3b shRNA transduction (Figures 4(e) and 4(i)) in ox-LDL stimulated Tregs. These results suggest that ox-LDL-induced inhibition of FOXP3 expression is modulated by DNMT3b in Tregs.

### 3.5. DNMT3b Inhibition Affected the Contents of Inflammatory Cytokines in Ox-LDL Stimulated Tregs

**Figure 5:**
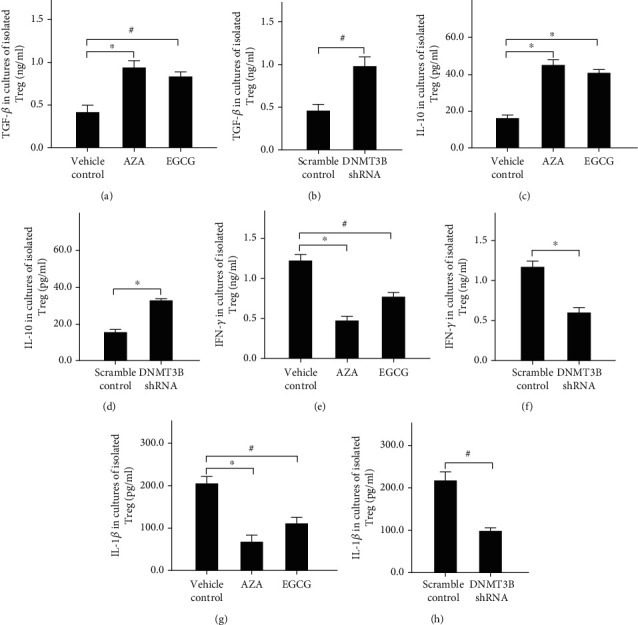
Cytokine level in ox-LDL cultures of isolated Tregs. ∗*P* < 0.01, #*P* < 0.05. (a) The concentrations of TGF-*β* by stimulating with AZA and EGCG in ox-LDL cultures of isolated Tregs. (b) The concentrations of TGF-*β* by transduced with DNMT3b shRNA in ox-LDL cultures of isolated Tregs. (c) The concentrations of IL-10 by stimulating with AZA and EGCG in ox-LDL cultures of isolated Tregs. (d) The concentrations of IL-10 by transduced with DNMT3b shRNA in ox-LDL cultures of isolated Tregs. (e) The concentrations of IFN-*γ* by stimulating with AZA and EGCG in ox-LDL cultures of isolated Tregs. (f) The concentrations of IFN-*γ* by transduced with DNMT3b shRNA in ox-LDL cultures of isolated Tregs. (g) The concentrations of IL-1*β* by stimulating with AZA and EGCG in ox-LDL cultures of isolated Tregs. (h) The concentrations of IL-1*β* by transduced with DNMT3b shRNA in ox-LDL cultures of isolated Tregs. Abbreviations: AZA: 5-aza-2′-deoxycytidine; DNMT3b: DNA methyltransferases 3b; EGCG: epigallocatechin-3-gallate; FOXP3: forkhead box P3; ox-LDL: oxidized low-density lipoprotein.

Compared with the vehicle control, the concentrations of TGF-*β* (Figure 5(a)) and IL-10 (Figure 5(c)) were increased by AZA and EGCG treatments in ox-LDL stimulated Tregs. DNMT3b silencing by shRNA transduction also significantly increased the concentrations of TGF-*β* (Figure 5(b)) and IL-10 (Figure 5(d)) in ox-LDL-treated Tregs. On the contrary, the levels of IFN-*γ* (Figures 5(e) and 5(f)) and IL-1*β* (Figures 5(g) and 5(h)) were significantly reduced after DNMT3b inhibition with inhibitors or DNMT3b-shRNA transduction, respectively. The reduction of inflammation factors was related to the increase of FOXP3 expression.

## 4. Discussion

In the present study, we found direct evidence that DNMT3b is involved in atherosclerosis. In addition, we have further revealed the possible mechanism. We found that Dnmt3b silencing ameliorated atherosclerosis was associated with increasing the Treg levels caused by reducing the methylation levels of *Foxp3*-TSDR and upregulated the expression of Foxp3 in Tregs.

The present study identifies that DNMT3b expression promotes atherosclerosis. It has been found that DNMT3b reduces the expression of genes by increasing the methylation level of the related genes, which affects the development of atherosclerosis. DNMT3b was found to be involved in atherosclerosis by regulating methylation levels of targeted genes in multiple atherosclerosis-related cell types. It was reported DNMT3b induced p53 hypermethylation and reduced its expression and promoted the proliferation of vascular smooth muscle cells [18]. DNMT3b can also reduce the expression of fibroblast growth factor 2 and promote atherosclerosis by increasing DNA methylation in endothelial cells [19]. Inhibition of scavenger receptor class B member 1 expression induced by DNMT3b accelerated atherosclerosis in foam cells [20]. These findings are in line with our observations that DNMT3b plays an important role in the development of atherosclerosis.

DNA methylation is mediated by three DNA methyltransferases, including DNMT1, DNMT3a, and DNMT3b [21]. According to the specific function, DNA methylation can be divided into two types: maintenance methylation and re-methylation. DNMT1 mainly mediates the maintenance of methylation, which is carried out according to the existing DNA methylation patterns. Conversely, DNMT3a and DNMT3b primarily perform as de novo methyltransferases which methylate DNA double strands that have not been methylated before [11, 22]. To the best of our knowledge, for the first time, our previous study identified a significant increase in DNMT3b (but not DNMT1 and DNMT 3a) expression was identified in Treg cells from patients with acute coronary syndrome [14]. Both expressions of DNMT3b and *FOXP3*-TSDR methylation levels were significantly increased in ox-LDL-treated human CD4+CD25+ T cells isolated from healthy volunteer [14]. In this study, we found that Dnmt3b silencing reversed the increase of *Foxp3*-TSDR methylation levels induced by WD in peripheral blood of ApoE^**−/−**^ mice. These results suggest that *Foxp3*-TSDR DNA methylation in Tregs is mediated by Dnmt3b.

**Figure 6:**
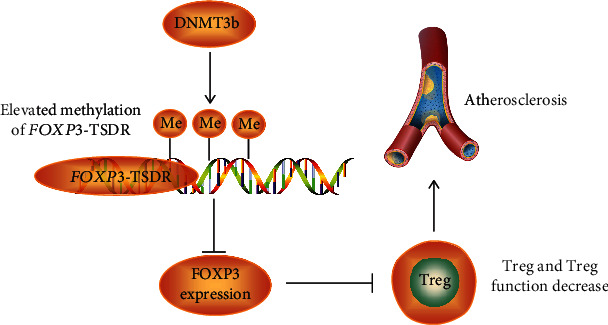
Schematic diagram of the aggravating role of DNMT3b in atherosclerosis. Abbreviations: DNMT3b: DNA methyltransferases 3b.

It has been demonstrated that DNA methylation–based regulation is crucial for controlling the expression of the FOXP3 [16, 23, 24]. The consistent FOXP3 expression is crucial for the maintenance of the suppressive properties of Tregs [25]. One of our previous studies has also established the association between *Foxp3*-TSDR DNA methylation and Treg: Treg levels are significantly inversely correlated with the methylation levels of *Foxp3*-TSDR in peripheral blood of ApoE^**−/−**^ mice [15]. Ox-LDL reduces the expression of Foxp3 by increasing the level of *Foxp3-*TSDR methylation [14]. In this study, our results showed that inhibition of DNMT3b expression levels significantly upregulated the expression of FOXP3 in Tregs. The continuous expression of FOXP3 is critical for the maintenance of Treg suppressive characteristics. It was believed that Tregs protected against the development and progression of atherosclerosis [26, 27]. Our previous studies also showed that WD induced Treg reduction and accelerated atherosclerosis [15]. Thus, we suppose that DNMT3b participated in accelerating atherosclerosis may be through regulating methylation levels of *Foxp3*-TSDR in Tregs and suppressing Treg function (Figure 6).

The present study provides a new insight into the aggravating role of DNMT3b in atherosclerosis. The current study provides a novel epigenetic mechanism of atherosclerosis. Moreover, our results proposed that *Foxp3*-TSDR hypermethylation may contribute significantly to the pathogenesis of atherosclerosis. Because proliferation of FOXP3^+^ Tregs can be induced by DNA methyltransferase inhibitor such as AZA and EGCG [17, 28–30], the epigenetic regulation of the *FOXP3* gene via DNMT3b shows potential therapeutic targets for atherosclerosis.

## 5. Conclusion

We demonstrated that DNMT3b accelerated atherosclerosis may be associated with *FOXP3*-TSDR hypermethylation, which is responsible for the subsequent down-regulation of FOXP3 expression and inhibition of Treg function.

## Data Availability

The data that support the findings of this study are available from the corresponding author on reasonable request.

## Supplementary Materials

Supplementary Materials Supplemental Figures: Figure S1. The diagram of animal experiments. Figure S2. The gating scheme of Treg in total peripheral blood of ApoE−/− mice. Figure S3. Construction, and transduction efficiency of pLVX-shRNA1-mDnmt3b. Figure S4. The lesion area of aortic root II. Supplemental Tables: Table 1. Sequences of Foxp3-TSDR used in this study. Table 2. Primers sequence of quantitative RT-PCR.
