# Supplementary material for: DNA Methyltransferase 3b Accelerates the Process of Atherosclerosis
Source: Oxid Med Cell Longev. 2022 Apr 5;2022:5249367. doi: 10.1155/2022/5249367 (PMC9005271; doi:10.1155/2022/5249367)
Supplement: Supplementary Materials — Supplemental Figures: Figure S1. The diagram of animal experiments. Figure S2. The gating scheme of Treg in total peripheral blood of ApoE−/− mice. Figure S3. Construction, and transduction efficiency of pLVX-shRNA1-mDnmt3b. Figure S4. The lesion area of aortic root II. Supplemental Tables: Table 1. Sequences of Foxp3-TSDR used in this study. Table 2. Primers sequence of quantitative RT-PCR. [file 5249367.f1.pdf]

Supplemental files

Contents

Ⅰ **Supplemental Figures**

Figure S1. The diagram of animal experiments.
Figure S2. The gating scheme of Treg in total peripheral blood of ApoE$^{-/-}$ mice
Figure S3. Construction, and transduction efficiency of pLVX-shRNA1-mDnmt3b
Figure S4. The lesion area of aortic root

Ⅱ **Supplemental Tables**

Table 1. Sequences of *Foxp3*-TSDR used in this study.
Table 2. Primers sequence of quantitative RT-PCR

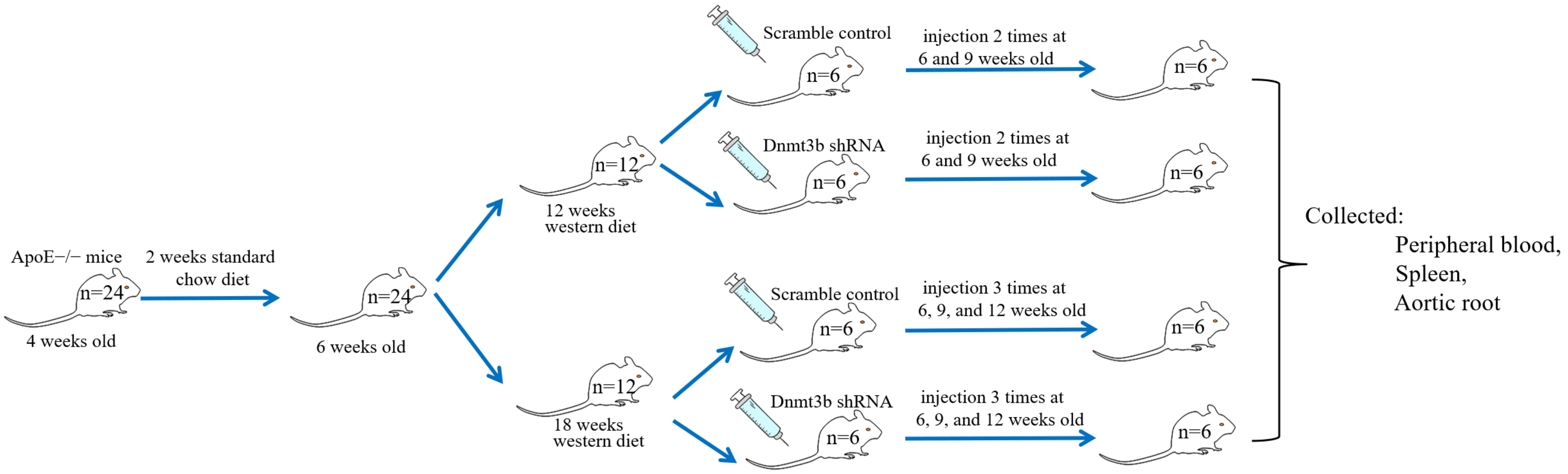

Figure S1. The diagram of animal experiments. Abbreviations: Dnmt3b, DNA methyltransferases 3b.

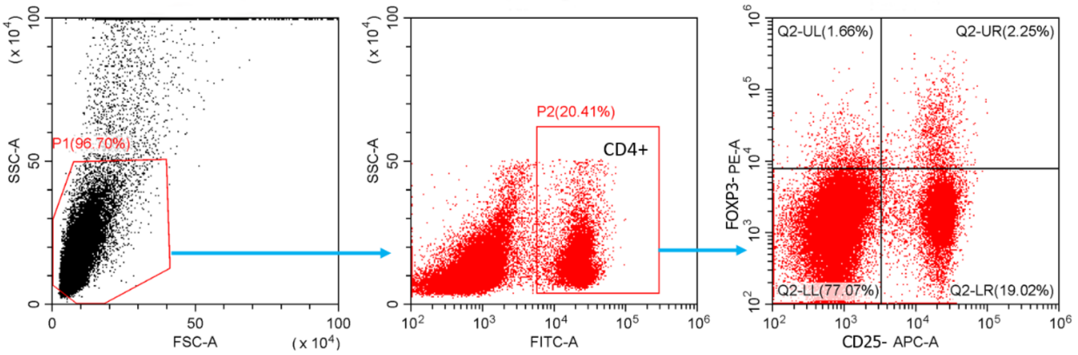

Figure S2. The gating scheme of Treg in total peripheral blood of ApoE$^{-/-}$ mice. We first gated the CD4^{+} T cells and then gated CD25^{+}Foxp3^{+} in CD4+ cells.

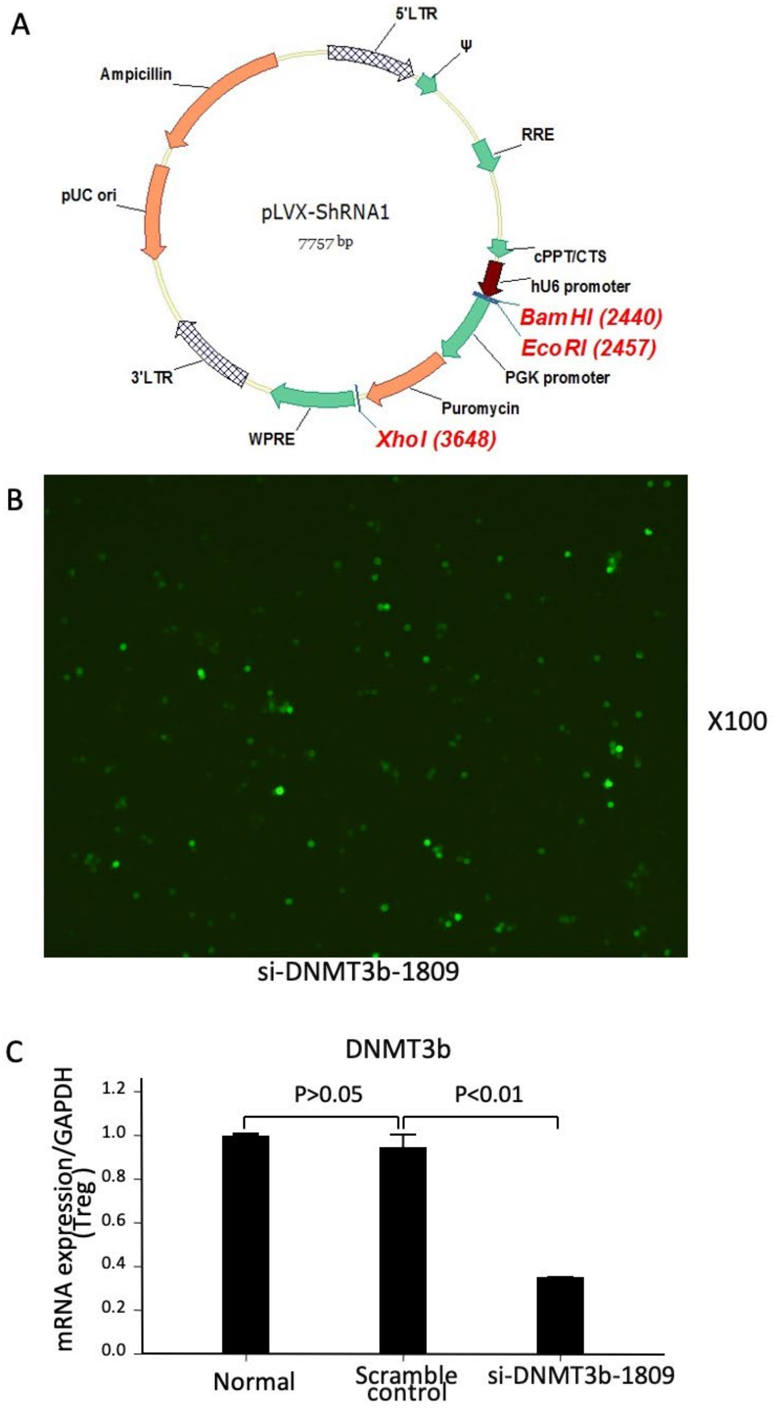

Figure S3: Construction, and transduction efficiency of pLVX-shRNA1-mDnmt3b. (A) structure of pLVX-shRNA1-mDnmt3b. (B) Transduction of viral vectors carrying *Dnmt3b* siRNA into isolated Tregs. (C) The mRNA level of Dnmt3b in spleen Treg of ApoE$^{-/-}$ mice transfected by viral vector carrying *Dnmt3b* siRNA.

Abbreviations: Dnmt3b, DNA methyltransferases 3b.

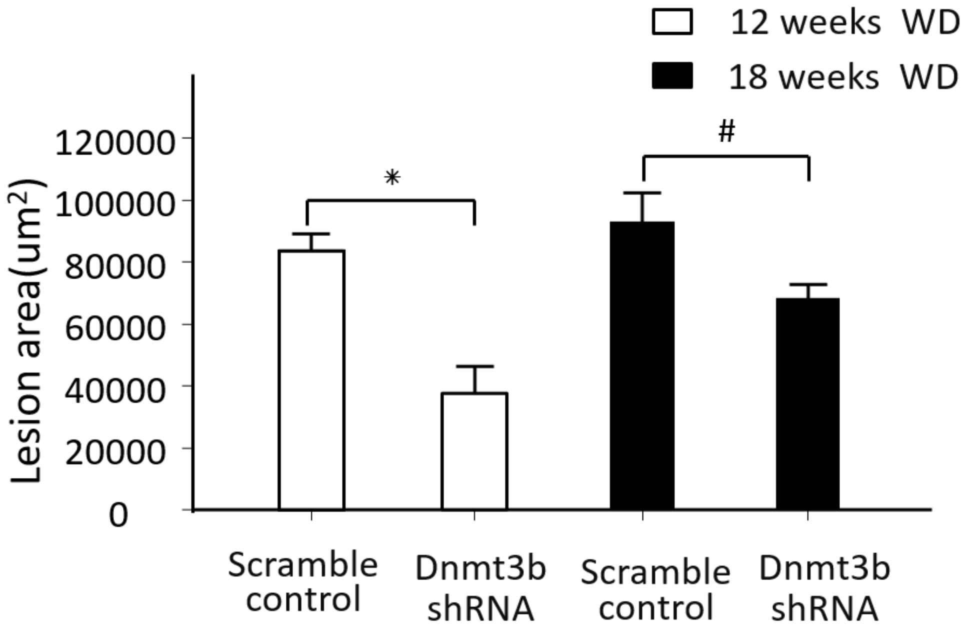

Figure S4: The lesion area of aortic root was measured by Oil-red O Staining. * *P*<0.01, # *P*<0.05.

Abbreviations: Dnmt3b, DNA methyltransferases 3b; WD, western diet.

Table S1 Sequences of *FOXP3*-TSDR used in this study

Assay type	Primer	Sequence
Mouse methylation specific qPCR	Forward	5′-TTGGGTTTTTTTGGTATTTAAGAAA-3′
	Reverse	5′-CCCTATTATCACAACCTAAACTTAACC-3′
Bisulfite sequencing of mouse *Foxp3*-TSDR		TTGGGTTTTTTTGGTATTTAAGAAAGATAGAATCG ATAGAATTTGGGTTTTGTATGGTAGTTAGATGGAC GTTATTTATTATATTCGTTAGCATTTATATTATTTTATT TGGGTTTATTCGGTTATAGGATAGATTAGTTATTTTT CGGAACGAAATTTGTGGGGTAGATTATTTGTTTTT TTTTTTTTTTTTTTGTTGTCGATGAAGTTTAATGTAT TCGGTCGTTATGACGTTAATGGTAGAAAAATTTGG TTAAGTTTAGGTTGTGATAATAGGG

CG: CpG site of bisulfite sequencing.

Abbreviations: Foxp3, Forkhead box P3; TSDR, Treg-specific demethylated region.

Table S2 Primers sequence of quantitative RT-PCR

Name	Primer	Sequence	Size
Homo DNMT3b	Forward	5'- GGGCATAAAGGTAGGAAAGT-3'	164bp
	Reverse	5'-TTCCGCCAATCACCAAGTCAA-3'	
Homo FOXP3	Forward	5'-CATTCCCAGAGTTCCTCCACA-3'	126bp
	Reverse	5'-CATTGAGTGTCCGCTGCTTC -3'	
Homo GAPDH	Forward	5'- TCAAGAAGGTGGTGAAGCAGG -3'	115bp
	Reverse	5'- TCAAAGGTGGAGGAGTGGGT -3'	
Mouse Dnmt3b	Forward	5'-TTGGTGCTCAAGGAGTTGGGTATTA-3'	217bp
	Reverse	5'-GGGCAGGATTGACGTTAGAGAGA-3'	
Mouse Foxp3	Forward	5'-CCTTTCACCTATGCCACCCTTATCC-3'	198bp
	Reverse	5'- TGCTCCCTTCTCGCTCTCCACT-3'	
Mouse Gapdh	Forward	5'-ATGGGTGTGAACCACGAGA-3'	229bp
	Reverse	5'-CAGGGATGATGTTCTGGGCA-3'	

Abbreviations: DNMT3b, DNA methyltransferases 3b; FOXP3, Forkhead box P3; GAPDH, glyceraldehyde-3-phosphate dehydrogenase; TSDR, Treg-specific demethylated region.
